# Comparison of Mortality and Hospital Readmissions Among Patients Receiving Virtual Ward Transitional Care vs Usual Postdischarge Care: A Systematic Review and Meta-analysis

**DOI:** 10.1001/jamanetworkopen.2022.19113

**Published:** 2022-06-28

**Authors:** Utkarsh Chauhan, Finlay A. McAlister

**Affiliations:** 1 Faculty of Medicine and Dentistry, University of Alberta, Edmonton, Alberta, Canada; 2 Division of General Internal Medicine, University of Alberta, Edmonton, Alberta, Canada

## Abstract

**Importance:**

Virtual wards (VWs) include patient assessment in their homes by health care personnel and offer ongoing assessment and case management via home, telephone, and/or clinic visits. The association between VWs and patient outcomes during the transition from the hospital to home are unclear; earlier reviews on this topic have often conflated telemonitoring programs with VW models.

**Objective:**

To evaluate the use of VW transition systems for community-dwelling individuals after medical discharge.

**Data Sources:**

English-language articles indexed in PubMed or Cochrane and published between January 1, 2000, and June 15, 2021.

**Study Selection:**

Randomized clinical trials comparing VW care with usual postdischarge care. Studies were stratified by diagnosis.

**Data Extraction and Synthesis:**

Following the Preferred Reporting Items for Systematic Reviews and Meta-analyses guideline, 2 reviewers independently identified studies and extracted data. DerSimonian-Laird inverse variance weighted random-effects models were used to compute relative risks (RRs) for dichotomous outcomes and mean differences for continuous outcomes.

**Main Outcomes and Measures:**

All-cause mortality, hospital readmissions, emergency department visits, health care costs, readmission length of stay, quality of life, and functional status.

**Results:**

Twenty-four randomized clinical trials (11 in patients with heart failure, 3 in patients with chronic obstructive pulmonary disease, 4 in patients at high-risk for readmission, and 6 in mixed patient populations) with 10 876 patients were included (20 more trials than earlier reviews). In patients with heart failure, VWs were associated with fewer deaths (RR, 0.86; 95% CI, 0.76-0.97) and fewer readmissions (RR, 0.84; 95% CI, 0.74-0.96). However, similar associations were not seen in randomized clinical trials enrolling patients with other diagnoses (RR, 0.93; 95% CI, 0.83-1.04 for mortality and RR, 0.96; 95% CI, 0.88-1.05 for readmissions). Across all studies, VWs were associated with fewer emergency department visits (RR, 0.83; 95% CI, 0.70-0.98) and shorter readmission lengths of stay (mean difference, −1.94 days; 95% CI, −3.28 to −0.60 days). Three of 7 studies that evaluated health care expenses reported statistically significant lower costs with VW transition systems.

**Conclusions and Relevance:**

Although postdischarge VW interventions appear to be associated with fewer subsequent emergency department visits, shorter readmission lengths of stay, and lower health care costs, fewer deaths and readmissions were seen only in trials enrolling patients with heart failure.

## Key Points

**Question:**

Is virtual ward transitional care following medical discharge to home associated with better outcomes compared with usual postdischarge care?

**Findings:**

In this systematic review and meta-analysis of 24 randomized clinical trials including 10 876 patients, virtual ward transition systems were associated with significantly fewer deaths and hospital readmissions in patients with heart failure. Across all diseases, virtual wards were also associated with fewer emergency department visits, shorter length of stay during readmissions, and lower health care costs.

**Meaning:**

In this study, although virtual ward interventions were associated with some better outcomes and lower costs, fewer deaths and readmissions were seen only in trials enrolling patients with heart failure.

## Teaser

The systematic review and meta-analysis evaluates randomized clinical trials on the use of virtual ward intervention compared with usual postdischarge care for patients following hospitalization.

## Introduction

Up to one-fifth of patients discharged from the hospital are readmitted within 30 days and up to one-third are readmitted within 3 months.^1^ Although many approaches to reducing readmissions have been tested, the results have often been disappointing, and those that have achieved reduction have been beneficial only in certain contexts.^2^ The virtual ward (VW), a model first developed in England in 2006, uses a multidisciplinary case-management model to provide short-term transitional care to community-dwelling patients after hospital discharge to prevent unplanned readmissions.^3^ The VW differs from the hospital at home concept, which seeks to deliver inpatient type care in patient homes, by focusing on the period after hospital discharge as patients transition to community care. Virtual ward models include patient assessment within their home by skilled health care professionals, care coordination, and multidisciplinary team-based case management by combinations of telehealth, in-home, and/or clinic visits. Despite the theoretical appeal of the model, 2 systematic reviews identified that the evidence on the use of VWs is mixed and limited (most studies were small and reported context-specific initiatives), and benefits were only seen in studies of patients with heart failure (HF).^4,5^ However, most of the studies included in those 2 reviews (6 of 10 in one^4^ and 26 of 31 in the other^5^) evaluated telemonitoring programs (ongoing telephone or video follow-up with or without transmission of patient-recorded vital signs) without the key component of the 2006 VW model: patient assessment within their homes by skilled health care personnel. As telemonitoring has been shown to be efficacious for patients with HF in a number of randomized clinical trials (RCTs),^6^ whether VW transitional care models are beneficial after hospital discharge remains unknown.

We conducted a systematic review and meta-analysis to evaluate the use of VW interventions in a wide range of patient outcomes after discharge, including mortality, hospital readmission, emergency department visits, health care costs, quality of life, and functional status. We compared outcomes stratified by characteristics of the patient population enrolled. Patient populations were classified a priori as HF, chronic obstructive pulmonary disease (COPD), high risk for readmission, and mixed medical diagnoses.

## Methods

### Data Sources and Searches

We searched the PubMed database for English full-text articles published between January 1, 2000, and June 15, 2021. The search algorithm incorporated common terms for postdischarge transitional care interventions of interest, including *virtual ward*, *hospital at home*, and *transitional care*, and several terms addressing outcomes such as *mortality*, *readmission*, and *cost* (eMethods in the Supplement). This study followed the Preferred Reporting Items for Systematic Reviews and Meta-analyses (PRISMA) reporting guideline. We hand-searched the reference lists of included articles and relevant reviews from the Cochrane Library.

### Study Selection

Publications were eligible for inclusion if they were studies in adult that compared a postdischarge VW in the community (involving at least 1 home visit by a health care practitioner after discharge with ongoing home visitations, care coordination, and daily case management with a multidisciplinary team) with usual postdischarge care in RCTs, studied patients discharged from a medical ward to home (as opposed to those needing postsurgical rehabilitation or were discharged to a skilled nursing facility), and evaluated outcomes such as all-cause mortality, hospital readmission, emergency department visits, length of stay, health care costs, quality of life, or functional status. Two reviewers (U.C. and F.A.M.) independently screened abstracts and full-text articles for inclusion. Systematic review software (Covidence, version; [Covidence]) was used to blind reviewer voting, identify conflicts, and support reaching consensus.^7^

### Data Extraction and Quality Assessment

One reviewer (U.C.) extracted information about study and patient characteristics, elements of each VW intervention, outcomes, and study quality, using the Cochrane Risk of Bias Tool,^8^ with confirmation by the second reviewer (F.A.M.). Conflicts were resolved via consensus.

### Statistical Analysis

We conducted our primary meta-analyses by categorizing data by patient population (HF, COPD, high risk for readmission, or mixed medical diagnoses). For dichotomous outcomes, we followed the intention-to-treat principle to compute relative risks (RRs) and used DerSimonian-Laird random-effects models with inverse-variance weights.^9^ For continuous outcomes, we computed mean differences using an inverse variance random-effects model. Results included in the meta-analyses correspond to outcomes reported for the maximum length of follow-up time in each study.

Studies that evaluated cost, quality of life, and functional status were too heterogeneous in their assessment methods and outcome measures to permit formal pooling; these outcomes are reported by the proportion of trials that found statistically significant benefit (reduced cost, increased quality of life) in the intervention group. We originally planned to conduct a meta-regression to see whether efficacy varied by type of VW care (including but not restricted to frequency and intensity of home visits); however, there was insufficient detail in the published studies or from authors we contacted to permit this analysis. Although we had also planned to evaluate for efficacy differences across geographic regions, there were too few studies in each region and too much heterogeneity in the results even within regions for this analysis to be done. All statistical analysis and forest plot synthesis was performed using Cochrane Review Manager, version.^10^ Two-sided significance testing was conducted with the significance threshold set at *P* < .05.

## Results

### Overview

We identified 1008 unique citations, of which 24 RCTs (10 876 participants; 23 RCTs with low risk of bias) met eligibility criteria (eFigure 1 in the Supplement). Overall, 11 studies reported nursing care–dominant VW interventions and 13 used multidisciplinary team care.

Ten of the RCTs investigated patients with HF,^11,12,13,14,15,16,17,18,19,20,21^ 3 evaluated patients with COPD,^22,23,24^ 4 investigated patients with high risk of readmission (either by LACE [risk scoring criteria based on length of stay, acuity of the admission, comorbidities, and emergency department use 6 months before admission] scoring or local hospitalization/complexity metrics),^25,26,27,28^ and 6 RCTs included patients with a mix of diagnoses.^29,30,31,32,33,34^ Five of the RCTs were conducted in the US, 5 in Asia, 4 in Australia, 5 in Europe, 3 in Canada, and 1 in South America, and 1 trial was multinational. Canadian studies contributed the largest weight to analyses (4579 participants; 43% of the RCT total). The Table provides characteristics of the included RCTs. Risk of bias assessments are reported in the eTable in the Supplement.

**Table.** Overview of Study Characteristics

Source	Sample size, No.	Study population (location)	Mean age	Key components of cohorts	Outcomes assessed (timeframe)	Quality score^a^
**HF population**						
Stewart et al,^11^ 2002	297	Patients with HF aged ≥55 y discharged to home (Australia)	75 y	Intervention: structured home visit within 7-14 d of discharge by nurse and pharmacist or cardiac nurse for physical examination, treatment adherence, education; care referrals and reports sent to primary care physician and cardiologist; nurse telephone follow-up for 6 mo; control: usual care (duration of intervention: 6 mo)	Primary (3 y): Frequency of hospital readmission and death; others (3 y): event-free survival (hospital readmission and death), mortality, hospital readmission, length of stay, type of hospital admission (elective/unplanned), health care costs, cost per life-year saved	6
Naylor et al,^12^ 2004	239	Patients with HF aged ≥65 y (Philadelphia, Pennsylvania)	76 y	Intervention: APN home visit within 24 h of admission, daily during hospitalization, and ≥8 home visits (initial visit <24 h postdischarge); additional APN visits based on patient needs; APN accessible by telephone and collaborated with patient’s MD; rehospitalization did not interrupt transitional care; APNs masters-prepared nurses who participated in a 2-mo HF training program; control: routine care from admitting hospital including site-specific heart failure management and discharge planning (duration of intervention: 3 mo)	Primary (6 mo): composite of hospital readmission or death; others (12 mo): hospital readmissions, total hospital days, quality of life, functional status, cost, satisfaction with care	7
Kwok et al,^13^ 2008	105	Patients aged ≥60 y with HF and 1 hospital admission for HF in the past 12 mo discharged from hospital (Hong Kong)	78 y	Intervention: community nurse visit before discharge, home visit within 7 d postdischarge, weekly for 4 wk, then monthly; visits provide physical examination, education, and medication adherence support; nurse liaised closely with hospital specialist and was accessible to patients during working hours; control: usual medical and social care (duration of intervention: 6 mo)	Primary (6 m): rate of hospital readmission; others (6 mo): No. of hospital readmissions, functional status, public health care and personal care costs	5
Leventhal et al,^20^ 2011	42	Patients admitted to internal medicine service with acute HF decompensation discharged to home (Basel, Switzerland)	77 y	Intervention: 1 home visit by an HF nurse 1 wk postdischarge and 17 telephone calls in decreasing intervals over 12 mo; specialized nursing plan and education kit given to patients; nurse coordination with primary care physician and consultation with study internist/cardiologist as needed; control: usual care and routine discharge measures; follow-up by primary care physician (duration of intervention: 12 mo)	Primary (6 mo): mortality, hospital readmission (HF-related and all-cause); others (6 mo): quality of life; length of stay for HF decompensation	5
Stewart et al,^14^ 2012	280	Patients with HF discharged with a recent history of ≥1 admission for HF (Australia and Cleveland, Ohio)	71 y	Intervention: home visit by a CHF nurse within 7-14 d postdischarge; visit included clinical and pharmacological assessment, counseling, and liaison with the patient’s family physician; control: received similar care at a nurse-led specialist clinic with access to multidisciplinary team including pharmacist and cardiologist; care included telephone follow-up and additional home visits (duration of intervention: up to 18 mo)	Primary (12-18 mo): composite of hospital readmission or mortality; others: hospital readmission (18 mo), mortality (18 mo), event-free survival (18 mo); pharmacologic therapy (12 mo); quality of life (12 mo); health care costs (18 mo)	6
Tsuchihashi-Makaya et al,^15^ 2013	168	Patients with HF discharged to home (Hokkaido, Japan)	76 y	Intervention: home visit by nurses within 14 d of discharge for symptom monitoring, education, and counseling; then every 2 wk until 2 mo postdischarge; telephone follow-up by nurse until 6 mo postdischarge; nurse consulted multidisciplinary team including MD and pharmacist during intervention period; control: usual care and follow-up; routine management by cardiologist with no extra follow-up by an HF nurse or multidisciplinary team (duration of intervention: 6 mo)	Primary (1 y): psychological status (depression and anxiety); others (1 y): quality of life, mortality, hospitalization for HF	4
de Souza et al,^16^ 2014	252	Patients with LVEF ≤45% and clinical HF discharged to home (Brazil)	62 y	Intervention: nurse-led intervention with 4 home visits (<10 d, days 30, 60, and 120) combined with 4 reinforcement telephone calls; home visits included physical examination, education on self-care and medication; control: standard institution-specific HF management involving medical outpatient visits and general practitioner follow-up; no home visits or telephone contact (duration of intervention: 6 mo)	Primary (6 mo): composite of ED visits, hospital readmission, death; others (6 mo): ED visits, hospital readmission, mortality	6
Yu et al,^17^ 2015	178	Patients aged ≥60 y with HF discharged to home (Hong Kong)	79 y	Intervention: cardiac nurse predischarge visit to assess health status, self-care, and patient concerns; 2 weekly nurse home visits after discharge for physical examination, education, and community support referral; nurse telephone calls 1 wk after second home visit, every 2 wk for 3 mo, then every 2 mo for 6 mo for counseling, advice, and CHF symptom monitoring; patients had telephone access to nurse during working hours; control: brief instructions on medication at discharge; specialist visit arranged for 4-6 wk postdischarge; no structured educational or supportive postdischarge care (duration of intervention: 9 mo)	Primary (9 mo): event-free survival (time to hospital readmission or death), hospital readmission, mortality; others (9 mo): length of stay, self-care, quality of life	6
Wong et al,^21^ 2016	84	Patients discharged with end-stage HF (Hong Kong)	78 y	Intervention: predischarge assessment by NCM; week 1 home visit by NCM and nursing students; subsequent follow-up by students at week 3; NCM telephone contact at weeks 2 and 4; monthly home visits and telephone follow-ups by NCM until 12 wk; control: usual care and 2 placebo telephone calls consisting of light conversation unrelated to medical management (duration of intervention: 3 mo)	Primary (1 and 3 mo): hospital readmission; others (1 mo): symptom intensity, functional status, quality of life, satisfaction with care	6
Huynh et al,^19^ 2019	412	Patients with HF hospital discharge (Australia)	74 y	Intervention: discharge home visit with transition coach, HF nurse, and cardiologist; transition coach telephone calls (1 within 3 d, another during week 2 of discharge); cardiac nurse home visits during first and second week; additional nurse telephone contact for patients as needed; control: standard disease management programming and a follow-up telephone call within 1 mo postdischarge	Primary (1 and 3 mo): composite of hospital readmission or death; others (1 and 3 mo): hospital readmission, mortality, results stratified by predicted risk score (previously validated)	6
Van Spall et al,^18^ 2019	2494	Patients hospitalized for HF discharged home (Ontario, Canada)	78 y	Intervention: needs assessment, multidisciplinary referrals, and HF self-scare education by nurse navigator at time of discharge; family physician follow-up within 1 wk; patients with LACE score ≥13 received nurse-led home visits (weekly, structured, in-person, and telephone assessments for 4-6 wk) before HF clinic visit; control: transitional care at the discretion of clinicians; 1 hospital included nurse-provided education and a home visit to select patients; 8 hospitals had access to heart function clinics, 2 did not (duration of intervention: 0.25-1.15 mo)	Primary: composite of hospital readmission, ED visit, or death (3 mo), composite of hospital readmission or ED visit (1 mo); others: individual components of primary outcomes, quality of transition and life (1.5 mo), quality of life and quality-adjusted life-years (6 mo)	
**COPD population**						
Hermiz et al,^22^ 2002	177	Patients discharged from hospital or ED with COPD (Sydney, Australia)	67 y	Intervention: 2 home visits (<1 wk, 1 mo) by community nurse for assessment, education, and referrals as needed; GP telephone contact made based on nurse discretion; control: discharge to GP care and possible specialist follow-up; no routine nurse or other community follow-up (duration of intervention: 1 mo)	Primary (3 mo): hospital visits and admission, quality of life, knowledge of illness, self-management, satisfaction, GP and nurse visits GP and nurse satisfaction	5
Casas et al,^24^ 2006	155	Patients admitted for COPD exacerbation discharged to home (Barcelona, Spain, and Leuven, Belgium)	71 y	Intervention: comprehensive discharge assessment, disease education, and individual care plans facilitated by specialized NCM; nurse availability maintained through web-based call center; in Barcelona, 1 joint visit of physician, nurse, and social worker with the case manager within 72 h of discharge; in Leuven, regular GP home visits; weekly telephone calls during first mo for patient education at both sites; control: outpatient control regimen at discretion of discharging attending physician; routine care including physician visits every 6 mo; no specialized nursing support, education, or call-center access; Physician visits did not significantly differ between intervention and control at follow-up (duration of intervention: 12 mo)	Primary (12 mo): hospital readmission; others (12 mo): readmission rate, survival without readmission, mortality, health care use	6
Aboumatar et al,^23^ 2019	240	Patients aged ≥40 y with ≥10 pack-year smoking history and given inpatient care for COPD discharged to home (Baltimore, Maryland)	65 y	Intervention: nurses with special training on COPD met with patient during hospital stay to support transition; nurses provided self-management support and addressed barriers to care for 3 mo after discharge via home visit or telephone; control: usual transitional care specific to study site including a general transition nurse to follow-up for 30 d postdischarge; nurse supported adherence to discharge plan and connection to outpatient care (duration of intervention: 3 mo)	Primary (6 mo): COPD-related hospitalizations and ED visits; others (6 mo): quality of life, mortality, time to death or first COPD-related hospitalization or ED visit	7
**High risk of readmission**						
Dhalla et al,^25^ 2014	1923	Patients discharged home from general internal medicine ward with LACE score ≥10 (Toronto, Canada); Reason for initial admission: 9% HF, 91% other	71 y	Intervention: multidisciplinary virtual ward; care coordination and provision through a combination of telephone, home, and clinic visits; daily oversight by multidisciplinary team; control: structured discharge summary given to patient and primary care physician, counseling and home care arrangements as needed, and recommendations for follow-up with primary care physicians or specialists; follow-up at a postdischarge clinic not routine at any site (at discharging clinician’s discretion) (duration of intervention: 0.5-2 mo)	Primary (1 mo): composite of hospital readmission or death; others (12 mo): composite of hospital readmission or death, hospital readmission, mortality, ED visits, nursing home admission	6
Hock Lee et al,^28^ 2015	827	Patients discharged from hospital with ≥2 unscheduled readmissions in the past 90 d and LACE score ≥10 (Singapore)	69 y	Intervention: multidisciplinary team including physician and APN conducted daily meetings to discuss patients; nurse telephone follow-up <72 h postdischarge, nurse home visit <2 wk after discharge; scheduled telephone calls once weekly; patients with unstable condition given urgent clinic appointments; control: usual medical care and copy of discharge summary; possible referral to primary care or outpatient specialists; no contact with study team for 3-mo interval (duration of intervention: 3 mo)	Primary (1 mo): hospital readmission; others (3 mo), hospital readmission; ED visits, patient satisfaction	5
McWilliams et al,^27^ 2019	1876 (699 in intervention arm did not receive intervention)	Patients discharged from hospital with high-risk for readmission based on local scoring method (North Carolina); Comorbidities: 24% HF, 21% COPD, 39% diabetes, 12% kidney failure	59 y	Intervention: transition services program including access to free-standing clinic, multidisciplinary team including internist, hospital follow-up (virtually from home or in clinic), medical reconciliation, and care coordination; minimum intensity of 1 in-person or virtual visit with a transition services medical professional; control: usual care including follow-up recommendations with primary care physicians and discharge summaries; home care and telephone calls arranged based on patient needs (duration of intervention: 1 mo)	Primary (1 mo): hospital readmission; others: hospital readmission (2 and 3 mo), ED visit (1 mo), ICU visit (3 mo), length of stay (3 mo)	6
Finkelstein et al,^26^ 2020	782	Patients discharged from hospital with medically and socially complex needs; medical complexity: 1 hospital admission <6 mo prior to index and ≥2 chronic conditions; social complexity: polypharmacy, barriers to accessing care, mental health condition, drug habit, homelessness (Camden, New Jersey); Mental health diagnosis at index admission: 30% depression, 44% substance abuse	65 y	Intervention: multidisciplinary team including nurses and social workers conducted home visits (mean, 7.6) and telephone calls (mean, 8.8); patients were accompanied to primary care physician and specialist visits; control: usual postdischarge care that may include home health care services or other outreach (services received were not measured) (duration of intervention: 3 mo [mean])	Primary (3 mo): hospital readmission; others (3 mo): proportion of patients with ≥2 readmissions, length of stay, hospital charges and payments received, mortality	7
**Mixed medical diagnoses/no risk stratification**						
Young et al,^29^ 2003	162	Patients with MI discharged home (Toronto, Canada); Medical history: 44% HF, 39% prior MI, 33% diabetes	79 y	Intervention: disease management program including 6 home visits from a cardiac-trained nurse, referral criteria to specialty care, communication with the family physician, patient education; control: referral to a noninvasive cardiac laboratory for diagnostic testing and cardiologist follow-up; possible referral to routine home care services (duration of intervention: 2 mo)	Primary: readmission days per 1000 follow-up days for angina, HF, and COPD; others: hospital readmission per 1000 follow-up days, ED visits, provincial claims for physician visits, diagnostic/laboratory services	6
Latour et al,^33^ 2006	208	Patients with ≥1 hospitalization in the previous 5 y discharged home (Amsterdam, the Netherlands); Medical diagnoses by ICD-9: 31% circulatory, 20% gastrointestinal, 17% respiratory, 7% endocrine, 5% infectious disease, 20% other	64 y	Intervention: NCM conducted home visit within 3-10 working d of discharge to assess patient complexity and functional status and build care plan in collaboration with medical supervisor; NCM home visits (minimum every 2 mo, varied by tailored care plan) and telephone contact; control: usual care, absent care management after discharge, care provided as per specialist and GP recommendation (duration of intervention: 6 mo)	Primary (6 mo): emergency readmission; others (6 mo): health care use, quality of life, psychological functioning	6
Rytter et al,^30^ 2010	331	Patients aged ≥78 y discharged from geriatric or internal medical ward (after minimum 2-d stay) to home (Denmark); Medical diagnosis: 25% cardiovascular (30% intervention, 19% control), 75% other	84 y	Intervention: structured home visit by GP and district nurse during week 1; appointment with GP in clinic or as home visit in weeks 3 and 8; visits include assessment, medication reconciliation, nursing support as needed; control: routine postdischarge care (duration of intervention: 2 mo)	Primary (6.5 mo): hospital readmission; others: GP adherence to hospital discharge recommendations (3 mo), health care costs (6.5 mo), functional status (3 mo), mortality (6.5 mo), patient satisfaction (3 mo), self-rated health (3 mo)	5
Stewart et al,^31^ 2015	624	Cardiac patients discharged to home with a cardiovascular diagnosis: CAD or diabetes but not HF (Australia); Clinical profile: 70% CAD, 26% diabetes, 17% peripheral vascular disease, 16% moderate/severe kidney dysfunction	66 y	Intervention: nurse home visit within 7-14 d postdischarge; nurse care included education, care plan formulation, referral to family physician; HF clinic visit at 18 mo postdischarge; supplemental home visits and telephone coaching as needed (mean, 5.1 vs 1.8); control: standard postdischarge and long-term management; no restrictions imposed on control group management, including cardiac rehabilitation (duration of intervention: 18 mo)	Primary (36 mo): composite of de novo HF hospitalization or mortality; others (36 mo) cardiovascular hospitalization, ED visits, length of stay, cardiac function	6
Buurman et al,^32^ 2016	674	Patients aged ≥65 y discharged from a medical ward with identification of seniors at risk-hospitalized patients score ≥2 (the Netherlands); Admission diagnosis: 29% infection, 14% gastrointestinal, 12% respirator, 11% cardiac	80 y	Intervention: CCRN handover before discharge from geriatric team; CCRN home or nursing home visits for medication reconciliation, assessment, and intervention; initial visit ≤2 d postdischarge, subsequent visits at 2, 6, 12, and 24 wk; control: usual care including comprehensive geriatric assessment during hospital admission; no CCRN involvement (duration of intervention: 6 mo)	Primary (6 mo): functional status; others (6 mo): mortality (1 and 6 mo), cognitive functioning, time to hospital readmission, time to discharge from a nursing home	6
Zimmerman et al,^34^ 2017	67 (in group 4; 222 total intention to treat)	Patients discharged from hospital to home with ≥3 chronic diseases (Omaha, Nebraska); Chronic conditions: 37% diabetes, 29% pulmonary disease	61 y	Intervention: education, self-management skills, medication reconciliation; group 1: intervention delivered by APRN-NP and certified nursing assistant team for 8 wk; group 2: same as group 1 but reduced intensity; group 3: Nursing coach intervention for 4 wk; if activation was low, patients were referred to APRN-NP and certified nurse assistant team for 4 additional wk; group 4: intervention at home or on telephone; 1 follow-up telephone call by nursing coach.; control: patients seen by discharge planner (social worker or nursing care coordinator) and given disease-specific discharge education; all patients received 1 follow-up telephone call 48 h postdischarge (duration of intervention: 2 mo)	Primary (2 and 6 mo): functional status, ED and inpatient costs, quality of life	2

Abbreviations: APN, advanced practice nurse; APRN-NP, advanced practice nurse/nurse practitioner; CAD, coronary artery disease; CCRN, community care registered nurse; CHF, congestive heart failure; COPD, chronic obstructive pulmonary disease; ED, emergency department; GP, general physician/practitioner; HF, heart failure; *ICD-9*, *International Classification of Diseases*, *Ninth Revision*; ICU, intensive care unit; LACE, risk scoring criteria based on length of stay, acuity of the admission, comorbidities, and ED use within 6 months before admission; LVEF, left-ventricular ejection fraction; MI, myocardial infarction; NCM, nurse case manager; NP, nurse practitioner.

^a^ Quality graded using the Effective Practice and Organization of Care score with a 9-point scale; score greater than or equal to 3 indicates high quality; less than 3, low quality (details reported in the eTable in the Supplement).

### Mortality

**Figure 1.**
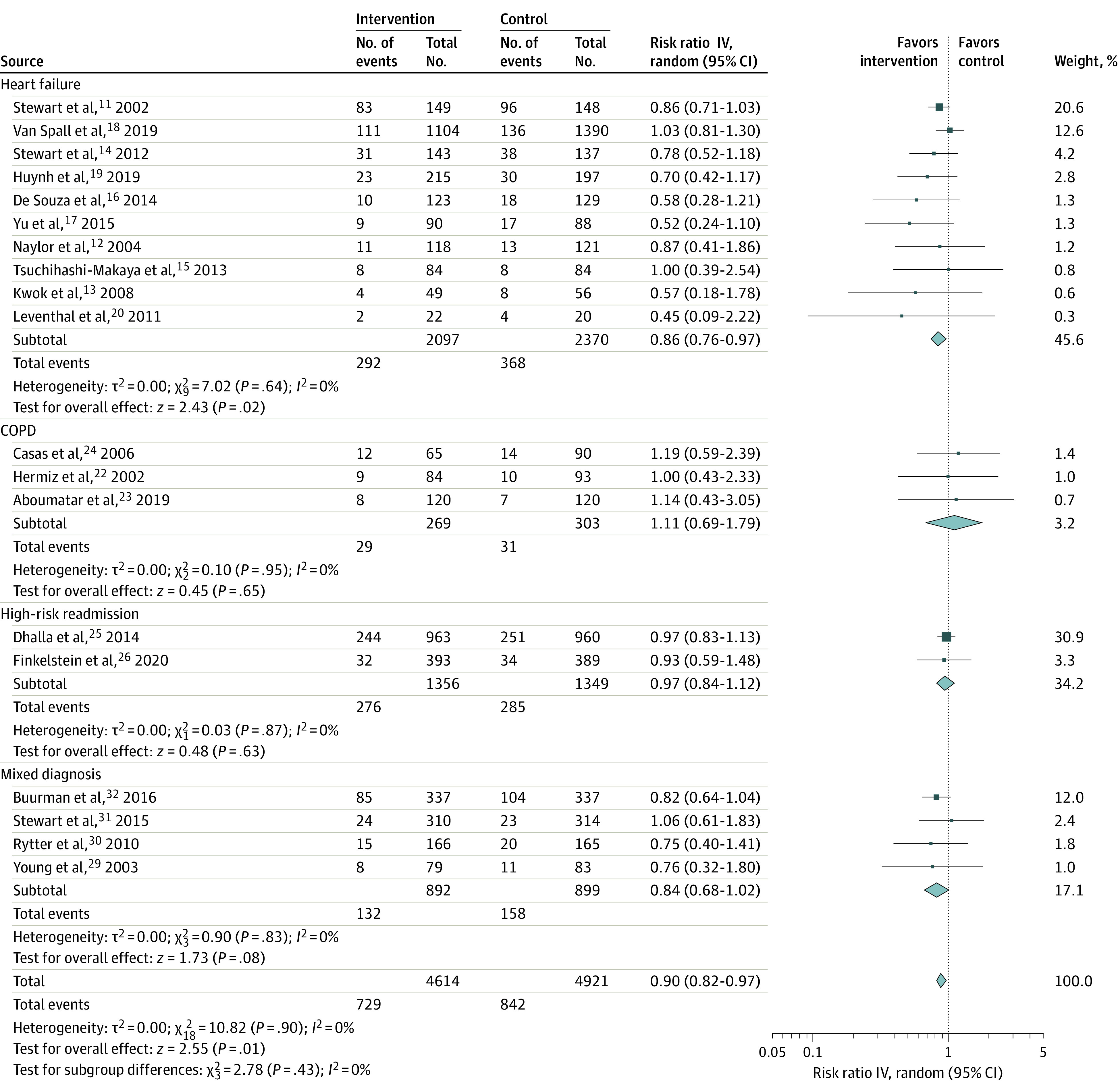
All-Cause Mortality After Discharge, Stratified by Patient Population COPD indicates chronic obstructive pulmonary disease.

Postdischarge VW transitional care was associated with fewer deaths in 10 RCTs enrolling patients with a primary diagnosis of HF (RR, 0.86; 95% CI, 0.76-0.97) (Figure 1).^11,12,13,14,15,16,17,18,19,20^ There was no significant difference in all-cause mortality in the non-HF RCTs (RR, 0.93; 95% CI, 0.83-1.04): 3 RCTs in patients with COPD (RR, 1.11; 95% CI, 0.69-1.79),^22,23,24^ 2 in patients considered high risk for readmission (RR, 0.97; 95% CI, 0.84-1.12),^25,26^ and 4 in patients with a variety of diagnoses (RR, 0.84; 95% CI, 0.68-1.02).^29,30,31,32^

### Hospital Readmission

**Figure 2.**
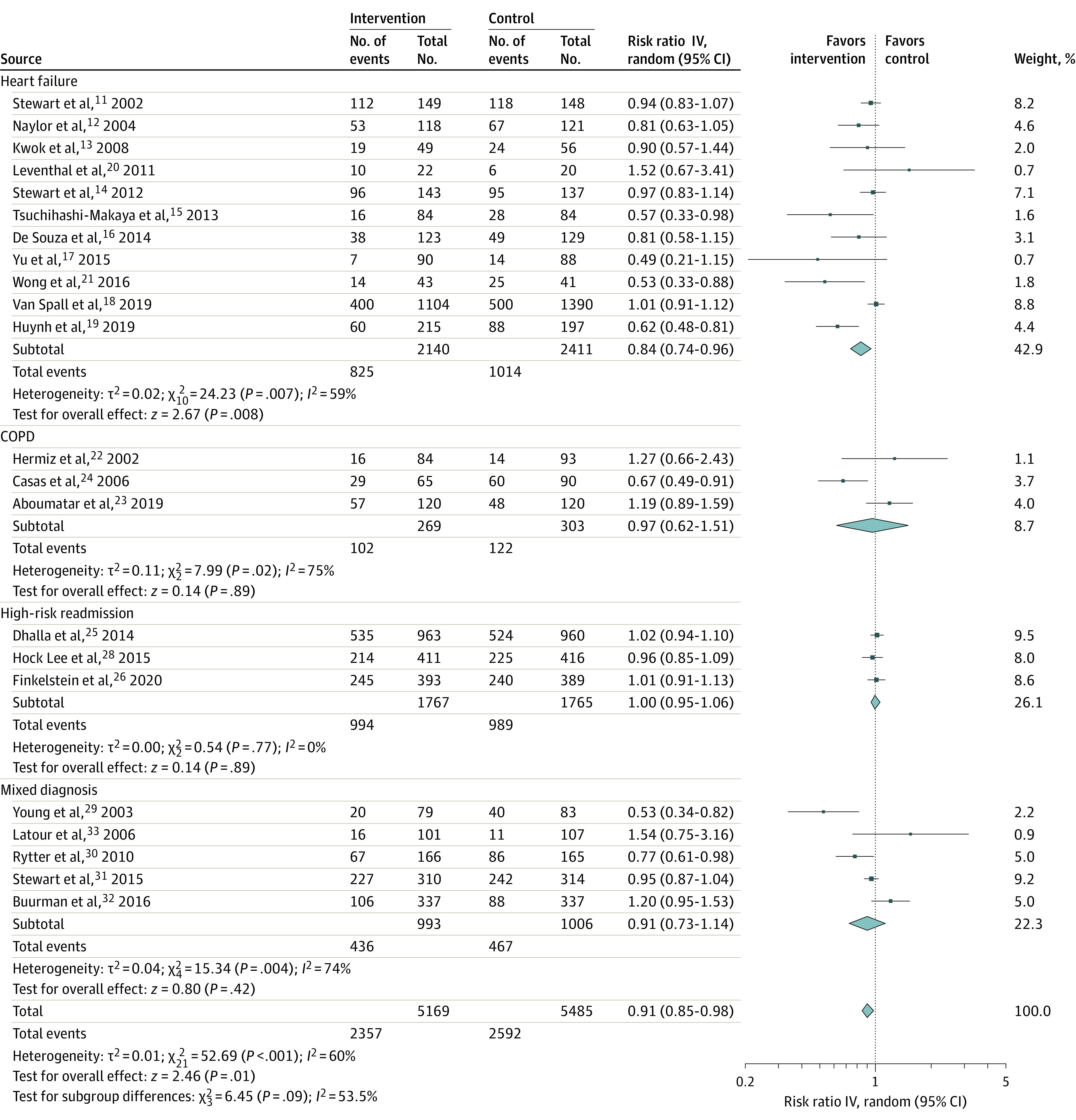
Hospital Readmissions, Stratified by Patient Population COPD indicates chronic obstructive pulmonary disease.

Twenty-three RCTs evaluated postdischarge hospital readmission.^11,12,13,14,15,16,17,18,19,20,21,22,23,24,25,26,27,28,29,30,31,32,33^ Virtual ward care was associated with fewer hospital readmissions in patients with a primary diagnosis of HF (RR, 0.84; 95% CI, 0.74-0.96) (Figure 2).^11,12,13,14,15,16,17,18,19,20,21^ However, the data from non-HF RCTs did not detect any significant difference in hospital readmissions (RR, 0.96; 95% CI, 0.88-1.05): 3 RCTs in patients with COPD (RR, 0.97; 95% CI, 0.62-1.51),^22,23,24^ 4 in patients considered high risk for readmission (RR, 1.00; 95% CI, 0.95-1.06; with McWilliams et al^27^ excluded from statistical aggregation owing to 75% participant attrition),^25,26,27,28^ and 5 in patients with a variety of diagnoses (RR, 0.91; 95% CI, 0.73-1.14).^29,30,31,32,33^

### Emergency Department Visits

**Figure 3.**
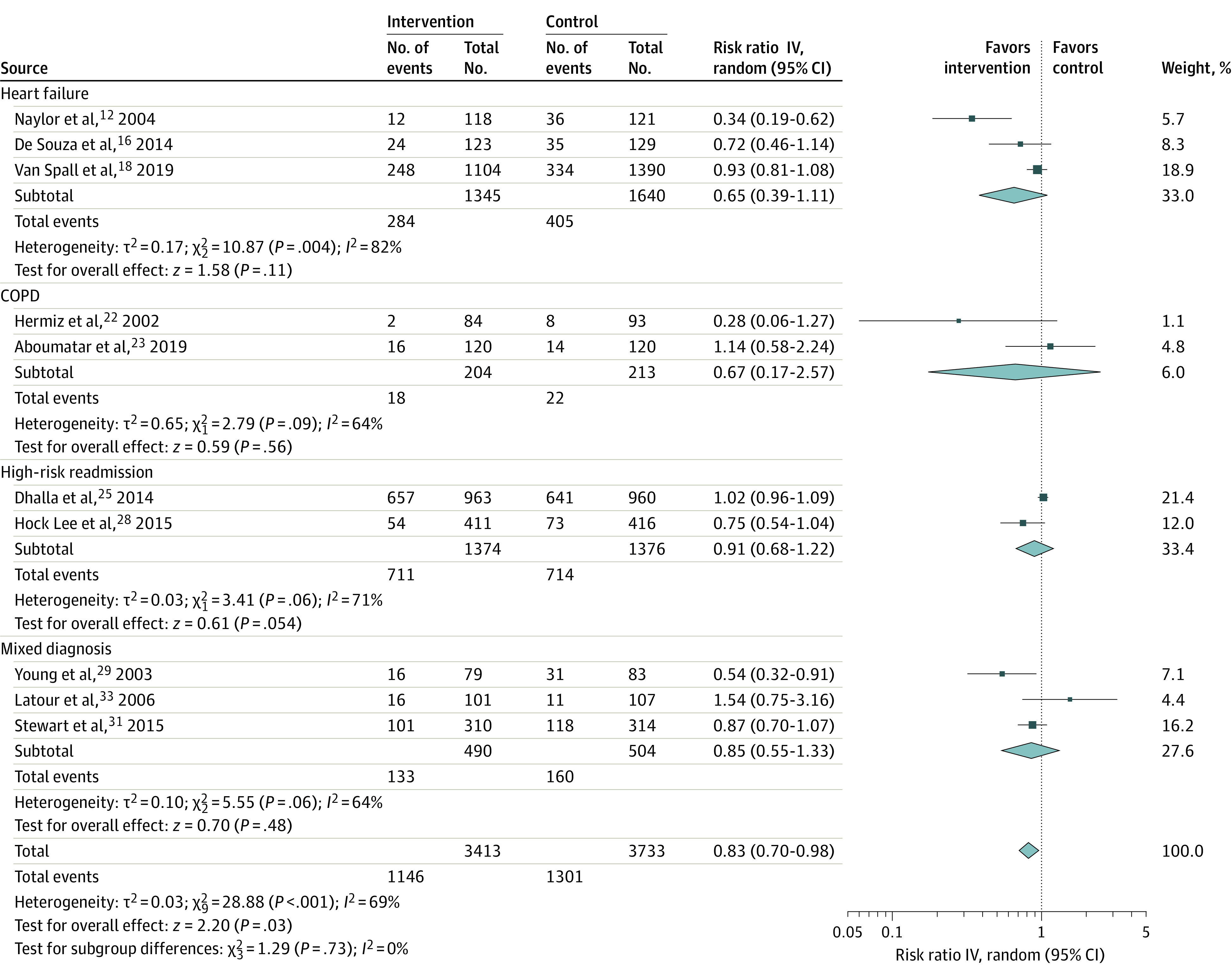
Emergency Department Visits After Discharge, Stratified by Patient Population COPD indicates chronic obstructive pulmonary disease.

Across all 11 trials, VW systems were associated with significantly fewer subsequent emergency department visits (RR, 0.83; 95% CI, 0.70-0.98) (Figure 3), although the results were very heterogeneous.^12,16,18,22,23,25,27,28,29,31,33^ Three of these trials were conducted in patients with a primary diagnosis of HF (RR, 0.65; 95% CI, 0.39-1.11),^12,16,18^ 2 in patients with COPD (RR, 0.67; 95% CI, 0.17-2.57),^22,23^ 3 in patients considered high risk for readmission (RR, 0.91; 95% CI, 0.68-1.22; with McWilliams et al^27^ excluded from statistical aggregation owing to 75% crossover to usual care),^25,27,28^ and 3 in patients with various mixed diagnoses (RR, 0.85; 95% CI, 0.55-1.33).^29,31,33^

### Health Care Costs

Seven RCTs compared total health care expenses between the VW intervention and usual care.^11,12,13,14,26,30,34^ Three RCTs reported significant cost-savings of $5000 to $10 000 per patient^11,12,14^ and 4 were cost-neutral.^13,26,30,34^ All RCTs reporting significant cost-savings enrolled HF cohorts.

### Length of Stay

**Figure 4.**
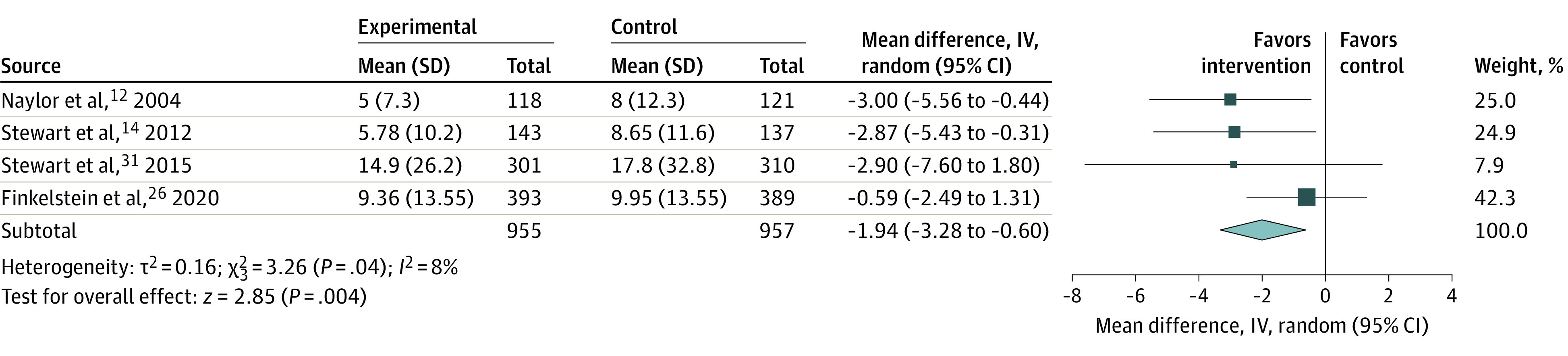
Length of Stay for Readmissions

Eight RCTs compared the mean length of stay for hospital readmissions following discharge between the VW intervention and usual care.^11,12,14,17,26,27,29,31^ Four RCTs reported the mean (SD) length of stay (mean difference in length of stay, −1.94 days; 95% CI, −3.28 to −0.60 days) (Figure 4).^13,15,25,30^ Data from 4 RCTs could not be statistically aggregated because they only reported *P* values: 2 RCTs^17,29^ reported a significant reduction in length of stay with *P* < .001 and 2 studies found no significant difference between groups.^11,27^ Overall, 4 of 8 studies reported a significant reduction in length of stay; 3 of these 4 only enrolled patients with HF.^12,14,17^

### Quality of Life

Eleven RCTs compared changes in quality of life over time between the VW intervention and usual care.^12,14,15,17,18,20,21,22,23,33,34^ Scoring systems included the Edmonton Symptom Assessment Scale, St George’s Respiratory Questionnaire, and Euroquol 5 Dimensions of Health. Six RCTs reported changes in quality-of-life scores with means (SDs) (standardized mean difference in score improvement, 0.11; 95% CI, −0.01 to 0.24) (eFigure 2 in the Supplement).^12,14,21,22,23,34^ Four RCTs could not be statistically aggregated because they only reported *P* values: 1 demonstrated a significant increase in quality of life,^15^ 1 showed a significant decrease,^17^ and 2 showed no or variable significance.^20,33^ One RCT had substantial attrition (45% attrition in the intervention group and 73% in the control group vs the intention-to-treat cohort) in quality-of-life reporting and was thus omitted from comparison.^18^

### Functional Status

Five RCTs compared changes in functional status over time between the VW intervention and usual care.^12,13,21,30,32^ Scoring systems included the 6-minute walk test, the Palliative Performance Scale, and the Katz Index for Activities of Daily Living. No RCTs reported significant differences in the improvement or exacerbation of functional status between groups.

## Discussion

We found that, in patients with HF, postdischarge VW interventions were associated with fewer deaths, fewer readmissions, and shorter hospital stays for patients who were readmitted. However, the association of VW transitional care programs with those outcomes in patients with other medical conditions who were discharged remain uncertain. All VW interventions that examined costs were either cost-neutral or cost-saving. The cost-saving VW interventions all enrolled HF cohorts and reported that reduced hospitalization and length of stay in the intervention group greatly offset the added expense of home visits and clinic visits. All 4 trials enrolling patients deemed at high risk for readmission did not demonstrate any benefit across several outcomes. This lack of benefit may indicate these interventions did not meet the needs of patients with complex conditions, a relative lack or delay in responsiveness for non-HF diagnoses (eg, COPD) to short-term changes in management, or perhaps scoring systems, such as LACE or social complexity scores, select for patients whose readmissions are not preventable. These patients may well require hospital care regardless of what is done and may not have factors that can be prevented or altered. Others have suggested that only a minority of readmissions are preventable.^35^ Thus, it would be useful for physicians to be prepared to identify patients who stand to benefit from specialized palliative services rather than VW transitional care programs.^36^

Unlike earlier reviews on this topic,^4,5^ we did not conflate telemonitoring interventions with VW interventions: the key distinguishing factor is that the VW model proposed in 2006 has as a central tenet that patients be assessed in their homes by trained health care personnel. Most trials included in earlier reviews of VW models evaluated telemonitoring case management and not VW models. In addition, those earlier reviews included trials in patients treated and released from emergency departments or RCTs evaluating comprehensive geriatric assessments at home rather than VWs for patients transitioning from the hospital to home. As a result, only 4 trials in those 2 earlier reviews evaluated VW models for patients discharged from medical wards to home; in this systematic review and meta-analysis, we identified 20 RCTs of VW models not included in earlier reviews.

The estimates of hospital readmission and emergency department visit reductions associated with VW were heterogeneous across studies, suggesting that the effect of VW interventions on these outcomes may be more sensitive to particular elements of each program. These outcomes are also prone to more potential bias than mortality: if clinicians are aware a patient is receiving intensive VW care at home, they may be less likely to suggest they present to an emergency department or, if they are seen in the emergency department, the clinician there may be less likely to admit them. Furthermore, the patient’s threshold to go to an emergency department will probably be influenced by whether they already have follow-up with a health care professional in place.

Half of VW interventions were associated with reduced length of stay when patients were readmitted compared with patients readmitted after usual postdischarge care. This is a notable finding as it suggests that the reduction in readmission rates for patients receiving VW is not just owing to a higher threshold for readmission; in that case, one would expect the patients being readmitted to be sicker and thus have longer lengths of stay. It is interesting to speculate whether direct collaboration between VW staff and inpatient clinicians improves information transfer and continuity, thereby enhancing the quality of inpatient care.

Quality-of-life reporting varied greatly across studies, as did the nature of the interventions, making it difficult to define an average result of VW interventions. Although some participants reported feelings of satisfaction and security with VW care and associated improvements in health, negative factors cited by others included an inability to disconnect from the health care system, the invasive nature of home visits, deeper internalization of a sick role and their dependency on the health care system, and greater awareness of symptoms influencing symptom-based quality-of-life metrics.

### Strengths and Limitations

This study has strengths. Our search strategy was broad and included hand searches of identified studies and review articles, we conducted our study as per PRISMA recommendations and focused on RCTs reporting objective outcomes, and we extracted several high-quality trials and stratified by patient population. Although some reviews limited investigation to the effects of VW interventions for patients with only 1 condition,^37^ we evaluated a wider array of outcomes and conditions to assess differential outcomes across patient populations.

The study has limitations. These include limited reporting of functional status, quality-of-life metrics, length of stay data, and lack of individual patient data that would permit meta-regression to evaluate for differences in efficacy over time or by more granular patient subgroups or specific elements of the interventions. In addition, although our interest was in all patients being discharged from medical wards, we found few RCTs of non-HF cohorts, particularly patients with COPD who make up a substantial proportion of readmitted patients. We are aware of VW programs for patients following stroke or surgical admissions and would encourage others to examine whether such programs demonstrate efficacy in those populations. This hypothesis should be tested rather than assumed based on our findings for non-HF populations in our systematic review and meta-analysis. With respect to our statistical analyses, we acknowledge that the DerSimonian-Laird random-effects model is limited in its consideration of study heterogeneity; it offers straightforward calculations but produces slightly narrower 95% CIs compared with more advanced statistical packages. In addition, VW programs in our study were heterogeneous in definition and intensity, which limits our ability to make specific recommendations about which VW elements are key for implementation.

## Conclusions

In this systematic review and meta-analysis, VW transitional care programs for recently discharged patients were associated with clear benefits in patients with HF, but their association with mortality or readmissions in patients with other chronic medical conditions or complex social situations remains uncertain and thus requires further research. Just as a single approach is not sufficient for most interventions in health care, it is clear from our data that not all patients benefit from VW transitional programs, even in the optimized setting of RCTs. Based on earlier studies, we believe that all patients discharged from medical wards should have an outpatient follow-up appointment within 2 weeks of discharge with a familiar physician (either their regular primary care clinician or a physician who cared for them during their hospitalization).^38,39,40^ We believe that VW transitional care programs should be targeted toward patients most likely to derive benefit; however, the difficulty is in identifying such individuals as even experienced clinicians and many risk prediction tools cannot accurately do so.^41,42^ Moreover, targeting VW interventions toward patients who need a high level of care is unlikely to be the best approach since fewer than half of such patients continue to need that level of care in consecutive years.^43^ As the comorbidity burdens of discharged patients increase, the proportion of patients who might potentially benefit from VW transitional care programs is likely to change and we would suggest that any VW transitional care program should be implemented with a robust evaluation component.

## Notes

Supplement. **eMethods.** PubMed Search Criteria **eFigure 1.** PRISMA Flow Diagram **eTable.** Risk of Bias **eFigure 2.** Quality of Life
