# Supplementary material for: Comparison of Mortality and Hospital Readmissions Among Patients Receiving Virtual Ward Transitional Care vs Usual Postdischarge Care: A Systematic Review and Meta-analysis
Source: JAMA Netw Open. 2022 Jun 28;5(6):e2219113. doi: 10.1001/jamanetworkopen.2022.19113 (PMC9240908; doi:10.1001/jamanetworkopen.2022.19113)
Supplement: Supplement. — eMethods. PubMed Search Criteria eFigure 1. PRISMA Flow Diagram eTable. Risk of Bias eFigure 2. Quality of Life [file jamanetwopen-e2219113-s001.pdf]

Supplementary Online Content

Chauhan U, McAlister FA. Comparison of mortality and hospital readmissions among patients receiving virtual ward transitional care vs usual postdischarge care: a systematic review and meta-analysis. *JAMA Netw Open*. 2022;5(6):e2219113. doi:10.1001/jamanetworkopen.2022.19113

eMethods. PubMed Search Criteria

eFigure 1. PRISMA Flow Diagram

eTable. Risk of Bias

eFigure 2. Quality of Life

This supplementary material has been provided by the authors to give readers additional information about their work.

© 2022 Chauhan U et al. *JAMA Network Open.*

eMethods PubMed Search Criteria

("virtual ward*"[tw] OR “community ward*”[tw] OR "hospital at home"[tw] OR hospital-at-home[tw] OR "community-based care"[tw] OR “community ward”[tw] OR “community care” OR “transitional care”[tw] OR “care transition*”[tw] OR “admission avoidance” [tw] OR “readmission avoidance”[tw] OR “prevent readmission*”[tw] OR telemanagement[tw] OR telemonitoring[tw] OR interdisciplinary[tw] OR multidisciplinary[tw] OR tele-homecare[tw])

AND

("standard of care"[tw] OR "usual care"[tw] OR “care as usual”[tw] OR "home care"[tw] OR homecare[tw] OR "Standard of Care"[Mesh] OR "Home Care Services" [Mesh] OR "Community Health Services"[Mesh])

AND

(discharge*[tw] OR "Patient Discharge"[Mesh])

AND

(outcome*[tw] OR readmission*[tw] OR hospitalization[tw] OR cost[tw] OR expens*[tw] OR "quality of life"[tw] OR “length of stay”[tw] OR death[tw] OR mortality[tw] OR morbidity[tw] OR exacerbat*[tw] OR emergen*[tw] OR "Patient Readmission"[Mesh] OR "Program Evaluation"[Mesh] OR "Treatment Outcome"[Mesh] OR "Costs and Cost Analysis"[Mesh] OR "Quality of Life"[Mesh] OR "Emergency Service, Hospital"[Mesh])

© 2022 Chauhan U et al. *JAMA Network Open.*

eFigure. PRISMA Flow Diagram

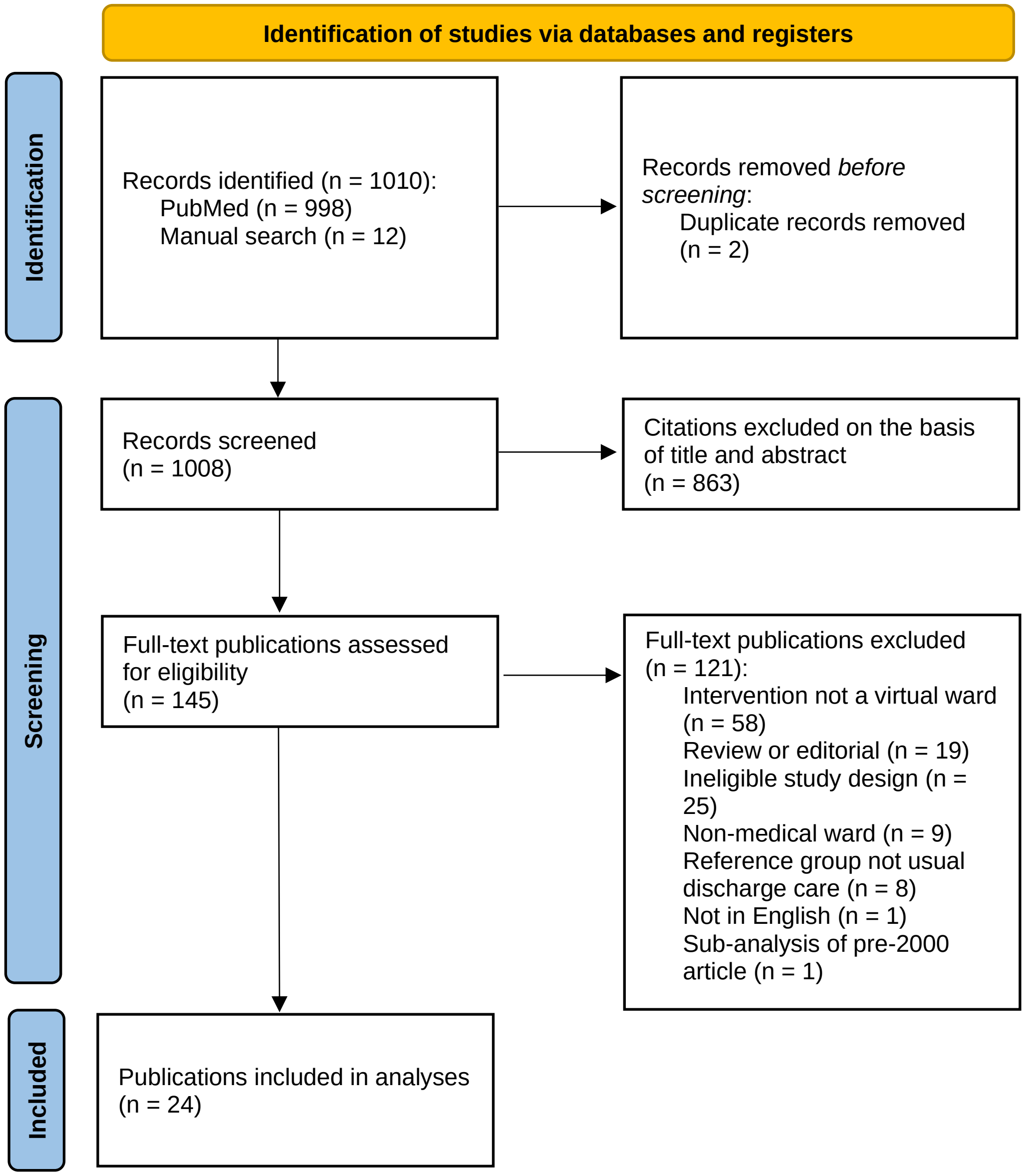

© 2022 Chauhan U et al. *JAMA Network Open.*

eTable. Risk of Bias

Study (Year) (Ref.)	Allocation sequence random?	Allocation concealed?	Baseline outcomes similar?	Baseline characteristics similar?	Plan for missing data/ incomplete measurement of primary outcome?	Outcomes assessed blind to intervention?	No contamination?	Free of selective outcome reporting risk?	No other bias? (including whether study was from single institution)	EPOC Group Risk of Bias criteria total (9 max)
Hermiz et al. (2002)[21]	Yes	No	Unclear	Yes	Unclear	Yes	Yes	Yes	No	5*
Stewart et al. (2002)[12]	Yes	No	Yes	Yes	Unclear	Yes	Yes	Yes	No	6
Young et al. (2003)[25]	Yes	No	Unclear	Yes	Yes	Yes	Yes	Yes	No	6
Naylor et al. (2004)[13]	Yes	No	Yes	Yes	Yes	Yes	Yes	Yes	No	7
Casas et al. (2006)[30]	Yes	No	Yes	Yes	Unclear	Yes	Yes	Yes	No	6
Latour et al. (2006)[31]	Yes	No	Yes	Yes	Unclear	Yes	Yes	Yes	No	6
Kwok et al. (2007)[14]	Yes	No	Unclear	Yes	Unclear	Yes	Yes	Yes	No	5
Rytter et al. (2010)[26]	Yes	No	Yes	No	Unclear	Yes	Yes	Yes	No	5
Leventhal et al. (2011)[29]	Yes	No	Unclear	Yes	No	Yes	Yes	Yes	No	5
Stewart et al. (2012)[15]	Yes	No	Yes	Yes	Unclear	Yes	Yes	Yes	No	6
Tsuchihashi-Makaya et al. (2013)[16]	Yes	No	Unclear	Yes	Unclear	Yes	Yes	No	No	4
de Souza et al. (2014)[17]	Yes	No	Yes	Yes	Unclear	Yes	Yes	Yes	No	6
Dhalla et al. (2014)[23]	Yes	No	Unclear	Yes	Yes	Yes	Yes	Yes	No	6
Lee et al. (2015)[34]	Yes	No	Yes	Yes	Unclear	Unclear	Yes	Yes	No	5
Stewart et al. (2015)[27]	Yes	No	Yes	Yes	Unclear	Yes	Yes	Yes	No	6
Yu et al. (2015)[18]	Yes	No	Unclear	Yes	Yes	Yes	Yes	Yes	No	6
Buurman et al. (2016)[28]	Yes	No	Yes	Yes	Unclear	Yes	Yes	Yes	No	6
Wong et al. (2016)[32]	Yes	No	Yes	Yes	Unclear	Yes	Yes	Yes	No	6
Zimmerman et al. (2017)[35]	Yes	No	Unclear	Unclear	No	Unclear	Yes	Unclear	No	2
McWilliams et al. (2018)[33]	Yes	No	Unclear	Yes	Yes	Yes	Yes	Yes	No	6
Aboumatar et al. (2019)[22]	Yes	No	Yes	Yes	Yes	Yes	Yes	Yes	No	7
Huynh et al. (2019)[20]	Yes	No	Yes	Yes	Unclear	Yes	Yes	Yes	No	6
Van Spall et al. (2019)[19]	Yes	No	Yes	Yes	Yes	Yes	Yes	Yes	No	7
Finkelstein et al. (2020)[24]	Yes	No	Yes	Yes	Yes	Yes	Yes	Yes	No	7

EPOC = Effective Practice and Organization of Care; * EPOC score out of 9 points; score $\geq$ 3 = high quality, < 3 = low quality

© 2022 Chauhan U et al. *JAMA Network Open.*

eFigure 2. Quality of Life

Study or Subgroup	Intervention Mean	Intervention SD	Intervention Total	Control Mean	Control SD	Control Total	Weight	Std. Mean Difference IV, Random, 95% CI	Year
Hermiz et al, 21, 2002	4.33	13.7	67	3	12.6	80	14.7%	0.10 [-0.22, 0.43]	2002
Naylor et al, 13, 2004	0.4	1.93	118	0.3	1.84	121	24.1%	0.05 [-0.20, 0.31]	2004
Stewart et al, 15, 2012	-0.136	0.363	143	-0.183	0.35	137	28.2%	0.13 [-0.10, 0.37]	2012
Wong et al, 32, 2016	1.06	1.995	43	0.11	2.735	41	8.3%	0.39 [-0.04, 0.83]	2016
Zimmerman et al, 35, 2017	0.076	0.122	34	0.055	0.135	33	6.7%	0.16 [-0.32, 0.64]	2017
Aboumatar et al, 22, 2019	2.81	28.9	88	-2.69	273	91	18.0%	0.03 [-0.27, 0.32]	2019
**Total (95% CI)**			**493**			**503**	**100.0%**	**0.11 [-0.01, 0.24]**	

Heterogeneity: Tau² = 0.00; Chi² = 2.24, df = 5 (P = 0.82); I² = 0%
Test for overall effect: Z = 1.78 (P = 0.07)

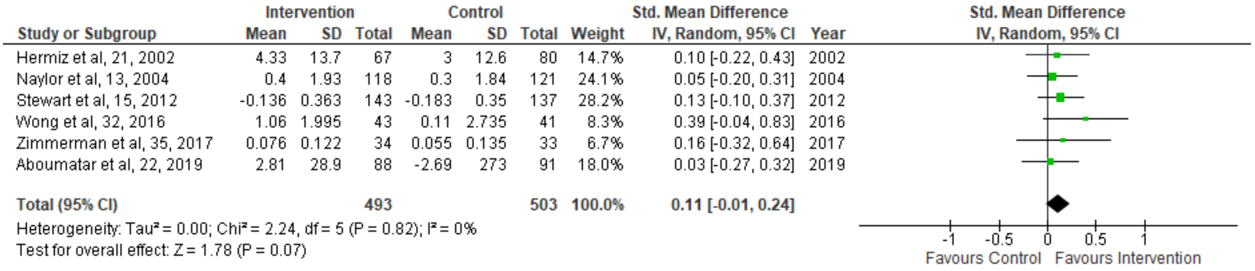

Closed diamonds represent the pooled odds ratio for each subgroup and 95% confidence interval. The final closed diamond aggregates all studies. Blue boxes represent individual study weights and center on the odds ratio of each study.

© 2022 Chauhan U et al. *JAMA Network Open.*
